# Fluoroscopy-guided aspiration of the acutely dislocated total hip arthroplasty: a feasible, high-yield, and safe procedure

**DOI:** 10.1186/s13244-024-01880-9

**Published:** 2025-01-10

**Authors:** Dyan V. Flores, Abdullah Felemban, Taryn Hodgdon, Paul Beaulé, George Grammatopoulos, Kawan S. Rakhra

**Affiliations:** 1 Department of Radiology, Radiation Oncology and Medical Physics Faculty of Medicine, University of Ottawa, Ottawa, ON Canada; 2 Department of Medical Imaging, The Ottawa Hospital, Ottawa, ON Canada; 3 Ottawa Hospital Research Institute, Ottawa, ON Canada; 4 Department of Radiology, King Faisal Specialist Hospital and Research Center, Riyadh, Saudi Arabia; 5 Division of Orthopaedic Surgery, The Ottawa Hospital, Ottawa, ON Canada

**Keywords:** Fluoroscopy, Total hip arthroplasty, Intervention, Image-guided hip aspiration, Dislocation

## Abstract

**Objective:**

To determine the feasibility, yield, and safety of fluoroscopic-guided aspiration of the acutely dislocated total hip arthroplasty (AD-THA).

**Materials and methods:**

IRB-approved, retrospective review of fluoroscopic-guided aspirations of AD-THA (January 2005–December 2023) was performed. Data from electronic charts and fluoroscopy images/reports were obtained. Positive yield was defined as spontaneous aspirate or saline rinse adequate for microbiology analysis. Sub-analysis by needle target (acetabular cup or femur) was performed for spontaneous aspiration rate, aspirate volume and fluoroscopy time. Differences between groups were analyzed with unpaired, *t*-test (2-tail) and between proportions with Fisher’s exact test, with significance *p* < 0.05.

**Results:**

Aspiration of 20 AD-THA in 19 patients (12 female, mean age (SD) of 73 years (16)) targeted the acetabular cup in 45% (9/20) or femur in 55% (11/20) of cases. Positive yield was obtained in 95% (19/20), with spontaneous aspirate in 75% (15/20) and saline rinse in 20% (4/20) of cases; in 5% (1/20), no diagnostic sample was obtained. Spontaneous aspirate mean volume (SD, range) for all cases was 8.3 mL (6.9, 0.2–25), and higher when targeting the acetabular cup 11.2 mL (6.9, 5–25) versus the femur 4.0 mL (4.4, 0.2–12) (*p* = 0.026). The rate of spontaneous aspiration was higher for the acetabular cup 100% (9/9) versus the femur 55% (6/11) (*p* = 0.038). The mean fluoroscopy time (SD, range) for all cases was 43 s (25, 19–102), and shorter for targeting the acetabular cup 32 s (16, 19–75) versus the femur 56 s (28, 28–102) (*p* = 0.034). No immediate complications occurred in all aspirations.

**Conclusion:**

Fluoroscopy-guided aspiration of AD-THA is a feasible, high-yield, and safe procedure. Targeting the acetabular cup results in a higher rate of spontaneous aspirate, larger aspiration volume, and lower fluoroscopy time.

**Critical relevance statement:**

Although technically more challenging, radiologists should feel confident aspirating the acutely dislocated total hip arthroplasty (AD-THA) under fluoroscopic guidance.

**Key Points:**

- Total hip arthroplasty (THA) infection can be evaluated with synovial fluid aspiration.
- Fluoroscopic-guided aspiration of the dislocated THA is feasible, high-yield, and safe.
- Targeting of the acetabular cup is recommended over the femoral prosthetic component.
- Acetabular cup targeting gives larger, spontaneous aspirates with lower fluoroscopy time.

**Graphical Abstract:**

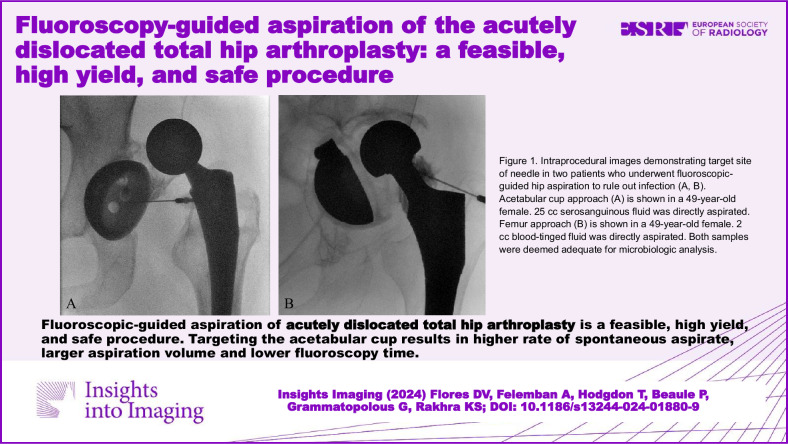

## Introduction

Dislocation is a known complication of primary and revision total hip arthroplasty (THA) [1]. It is challenging to manage and may be severely disabling to the patient, resulting in hardware loosening, migration or fracture, chronic hip instability, revision surgery, and/or severe patient distress and dissatisfaction [2]. Dislocation rates after primary THA range from 0.5 to 5% [3], while frequency rates following revision THA are even higher, ranging from 0.95 to 27% [4–7]. There are numerous risk factors for a dislocated THA, most commonly prior hip surgery, suboptimal surgical technique or component placement, soft tissue laxity, and patient behavior [8]. Although a less prevalent cause of dislocation, prosthetic joint infection (PJI) following THA is significant, with a reported incidence of 0.5–3% for primary THAs [9] and between 0.95 and 22% for revision THAs [3, 7].

Failure to recognize a PJI as a contributing cause to THA instability and dislocation can be devastating, resulting in increased morbidity and mortality due to prolonged hospitalization, extended antibiotic use, multiple revision surgeries causing progressive soft tissue and bone loss, and even death [10]. Thus, in the setting of THA dislocation, it is important to evaluate for possible infection with synovial fluid analysis factoring significantly in the overall diagnosis of PJI [11].

The standard of care at our institution is to perform image-guided aspiration by conventional fluoroscopy of a THA prior to any possible revision surgery and/or initiation of antibiotic treatment. Several studies have demonstrated the high efficacy of preoperative aspiration of the enlocated THA [12–14]. Ali and colleagues showed the sensitivity, specificity, positive and negative predictive values, and accuracy of hip aspirate cultures to be 0.82, 0.91, 0.74, 0.94, and 0.89, respectively, compared to intraoperative cultures [12].

However, on occasion, patients may present with a fixed, acutely dislocated total hip arthroplasty (AD-THA) where attempts at closed reduction have failed. In a dislocated prosthesis, the anatomy may be distorted, with disruption of normal imaging-based osseous and hardware relationships and landmarks. Extensive soft tissue swelling, limited mobility and malposition of the lower extremity and patient pain can make aspiration of the AD-THA more challenging than an enlocated hip. Additionally, there may be shifting of the femoral neurovascular bundle in an AD-THA potentially increasing the risk of bleeding or neuropraxia. There have not been any studies evaluating the efficacy of performing image-guided aspiration in the setting of a fixed, acutely dislocated total hip arthroplasty. The purpose of this study is to determine the feasibility, yield and safety of fluoroscopic-guided aspiration in the AD-THA.

## Materials and methods

### Subject selection

Institutional research ethics board approval was obtained for this retrospective study performed at a single, adult, tertiary university hospital. All fluoroscopy-guided aspirations of a dislocated THA were found by searching the institutional radiology reports database identifying all consecutive hip aspirations performed under fluoroscopy, between January 1, 2005, to December 31, 2023, followed by application of keyword search filter terms of “hip dislocation” or “dislocation.” Inclusion criteria were any patient with AD-THA confirmed on pre-procedure fluoroscopy images who underwent joint aspiration. Exclusion criteria were imaging evidence of reduction of the dislocated prosthesis before the aspiration, dislocation of a hip without THA, lack of adequate fluoroscopic images demonstrating needle placement site or lack of procedural report details on the nature of aspirate. Instances of aspirations in patients with multiple aspirations were all included.

### Joint aspiration technique

It is our institutional orthopedic preference to perform image-guided aspiration in all patients with hip arthroplasty dislocation, to exclude PJI prior to initiation of antibiotic treatment and/or any surgical relocation and possible revision surgery. Upon presentation to the emergency department with hip dislocation, patients will have reduction attempted under conscious sedation by the orthopedic service. However, should the reduction fail, the hip aspiration will still be performed, albeit on the fixed, dislocated THA.

Aspirations are performed with the patient supine and ipsilateral lower extremity positioned in neutral to slight internal rotation as tolerated, with a cushion under the knee giving slight flexion. The surface of the proximal neck of the femoral prosthesis is targeted from an anterior or slight anterolateral approach. Aseptic technique is employed, starting with the infiltration of the superficial soft tissues with buffered 1% lidocaine local anesthetic. A 20 gauge, styletted needle is then gradually advanced using step-and-shoot fluoroscopic guidance until there is firm engagement with the hardware. A 10 mL syringe is used to suction the synovial fluid, although if no aspirate is returned, the operator may adjust the needle and reattempt aspiration or advance the needle at their discretion to the surface of the acetabular cup. Upon engagement of the femoral or acetabular component, should no aspirate be returned, a wash is performed with 10 mL of sterile saline injected and then re-aspirated. A small volume of water-soluble contrast medium is injected confirming intraarticular positioning of the needle tip after the aspiration is performed. The fluid samples are transferred into sterile glass tubes and then sent to the onsite hospital laboratory for microbiological analysis. At our academic center with resident and fellow trainees, the standard of practice is that a staff radiologist directly supervises and assists trainees for complex aspirations such as the AD-THA.

### Fluoroscopic image and procedure analysis

Fluoroscopic images, along with the pre-procedure imaging were reviewed to document the direction of dislocation. Fluoroscopic images were used to determine the needle target site, either the acetabular cup or femoral component of the THA. The time of fluoroscopy (s) was recorded for each case. The procedural reports were reviewed for whether a spontaneous aspirate was obtained (with volume (mL)), if a saline rinse sample was obtained or if no sample was obtained. For the samples sent to the microbiology laboratory, the final reports were reviewed to confirm that they were adequate for a definitive microbiology report to be generated. Any immediate complications of bleeding or neuropraxia documented in the procedural report were recorded.

### Statistical analysis

Demographic data on age and gender, laterality of the hip aspirated and dislocation direction and subspeciality and/or level of training of the performing radiologist were tabulated. A positive yield was defined as a spontaneous aspirate or saline rinse sample obtained and deemed satisfactory for lab analysis based on microbiology report. The rate of spontaneous aspiration, mean volume (mL) of spontaneous aspirate and mean fluoroscopic procedural time (s) were calculated. Sub-analysis by needle target site (acetabular cup or femur) was performed for spontaneous aspiration rate, aspirate volume, fluoroscopy time and complications. Differences between groups were analyzed with unpaired, 2-tailed *t*-test and between proportions with Fisher’s exact test, with significance set at *p* < 0.05.

## Results

**Fig. 1:**
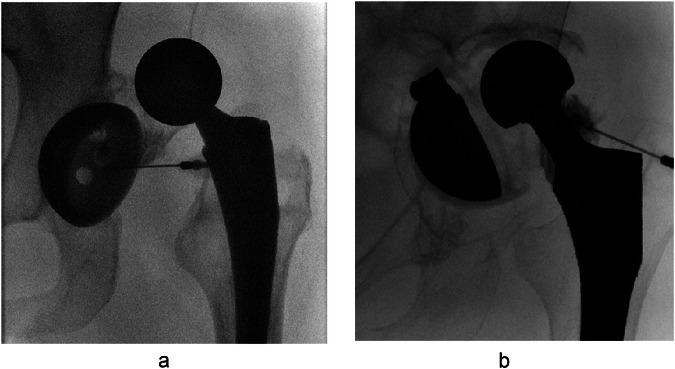
Intraprocedural images demonstrating target site of needle in two patients who underwent fluoroscopic-guided hip aspiration to rule out infection (**a**, **b**). Acetabular cup approach (**a**) is shown in a 49-year-old female. 25 cc serosanguinous fluid was directly aspirated. Femur approach (**b**) is shown in a 49-year-old female. 2 cc blood-tinged fluid was directly aspirated. Both samples were deemed adequate for microbiologic analysis

A total of 1735 hip aspirations were retrieved for the evaluation period, and after applying keyword filters, 41 aspirations remained, all of which had a THA. A further 21 cases were excluded as the THA was reduced at the time of aspiration. This resulted in 20 hips (12 left, 8 right) from 19 patients (12 females, 7 males), with mean age of 73 years (SD 16, range 37–94), included in the study. One female patient had two aspirations of the same hip performed on two separate admissions 2 years apart. The AD-THA were dislocated in the posterior (11/20), lateral (8/20) or anterior (1/20) directions. The aspirations were performed by fellowship-trained musculoskeletal radiologists (11/20), fellowship-trained body radiologists (3/20), or radiology residents/musculoskeletal radiology fellows (6/20) directly supervised by a staff radiologist. Aspirations targeted the femur in 55% (11/20) or acetabular cup in 45% (9/20) of cases (Fig. 1).

Positive yield was obtained in 95% (19/20) of procedures, with spontaneous aspirate in 75% (15/20) and saline rinse in 20% (4/20) of cases; in 5% (1/20) neither aspirate nor saline rinse was obtained. For spontaneous aspirate, the mean volume (SD, range) was 8.3 mL (6.9, 0.2–25), and when subdivided by needle target, significantly higher for the acetabular cup 11.2 mL (6.9, 5–25) versus the femur 4.0 mL (4.4, 0.2–12) (*p* = 0.026) (Table 1). The rate of spontaneous aspiration based on needle target was significantly higher for the acetabular cup 100% (9/9) vs. the femur 55% (6/11) (*p* = 0.038). The mean fluoroscopy time (SD, range) for all cases was 43 s (25, 19–102), and when subdivided by needle target site was significantly shorter for the acetabular cup 32 s (16, 19–75) vs. the femur 56 s (28, 28–102) (*p* = 0.034) (Table 1). No immediate complications of bleeding or neuropraxia occurred in any of the 20 aspirations.

**Table 1:** Rate of spontaneous aspirate, aspirate volume and fluoroscopy time based on needle target site

	Acetabular cup	Femur	*p*-value
Target site (%)	11/20 (55%)	9/20 (45%)	
Spontaneous aspiration rate (%)	9/9 (100%)	6/11 (55%)	0.038
Mean volume of spontaneous aspirate (mL) (SD, range)	11.2 (6.9, 5–25)	4.0 (4.4, 0.2–12)	0.026
Mean fluoroscopy time (s) (SD, range)	32 (16, 19–75)	56 (28, 28–102)	0.034

Positive yield was obtained in 19/20 of aspirations (15 spontaneous aspirate, 4 saline rinse) of which 4 reported positive growth of organisms. For spontaneous aspirate, 3 of 15 cases demonstrated growth of coagulase-negative *Staphylococcus* (2) and *Enterococcus faecalis* (1). For saline rinse, one of four cases demonstrated growth of coagulase-negative *Staphylococcus*. The one case for which no spontaneous aspirate nor saline wash sample could be obtained was in a 67-year male, with morbid obesity (body mass index 67).

## Discussion

Although a rare cause of THA dislocation, PJI may be a significant predisposing factor, requiring surgical relocation and revision surgery. Joint aspiration with synovial fluid sampling for infectious disease management and/or pre-revision surgery workup is an important procedure to exclude or confirm infection, identify the organism and perform antibiotic susceptibility testing [14]. Current literature supports preoperative joint aspiration in patients with risk of infection [12, 15, 16], and although there have been several published reports on image-guided joint aspiration of suspected infected enlocated THA, there are none in an AD-THA. Thus, we aimed to determine the efficacy of fluoroscopic-guided aspiration of the acutely dislocated total hip arthroplasty (AD-THA). The findings in this study demonstrate the feasibility of fluoroscopic-guided THA aspiration which is highly yielding (95%) and safe without any complication in the presented cohort.

The spontaneous aspiration volume was found to be significantly higher when the acetabular cup was targeted versus the femoral prosthesis. This may relate to the concave shape of the cup that can collect and hold fluid, especially when anteverted. This is in contradistinction to the neck or head of the femoral component that has rounded, convex surfaces over which fluid spills over into a more dependent location. Furthermore, accumulation or retention of fluid around the femoral neck requires integrity of the capsule that would be disrupted or deficient in the AD-THA. The overall spontaneous aspiration rate in this study, 75% for all cases and 100% for acetabular targeted cases is greater than reported in a study of 202 fluoroscopic-guided hip aspirations for suspected PJI where only the femoral neck was targeted [15]. Additionally, the mean spontaneous aspiration volume for the femoral targeted procedures was significantly lower, with two cases having ≤ 0.5 mL, too small to allow for part of the sample to be sent for cell count analysis at our institution where 1 mL minimum is required. This further supports targeting the acetabular cup, where in this study a spontaneous aspiration of ≥ 1 mL was obtained in all cases.

While studies have found US to be effective for both native hip and THA aspiration [17–19], our institution utilizes fluoroscopy for all joint aspirations and injections due to greater fluoroscopy resource availability and radiologist preference, confidence, and expertise. Although US allows for direct visualization of soft tissue findings of infection, such as effusion or fluid collections, this advantage may be diminished in the setting of AD-THA. First, it is well-recognized that US is limited in large or obese patients due to US beam attenuation [20], thereby limiting image quality. A morbidly obese patient may even warrant greater transducer pressure limiting the dexterity of the operator in simultaneously maneuvering needle and transducer. Second, extensive local soft tissue edema and hemorrhage associated with acute dislocation may negatively impact tissue thickness and attenuation, thereby further deteriorating the visibility of landmarks and, consequently, the needle and target. Finally, US relies on a localized “field of view” showing specific anatomic landmarks for a precise aspiration, and AD-THA places these landmarks at risk for significant or even complete obscuration. Although fluoroscopy may not allow for direct visualization of soft tissue structures, it does provide excellent continuous, real-time global visualization of the joint, hardware, needle and essential landmarks that can facilitate a safe and efficient procedure. While fluoroscopy has limited soft tissue visualization, administration of contrast can confirm intraarticular needle position and demonstrate extension or communication of infection from the joint to the extra-articular tissues. An advantage of performing aspiration under fluoroscopy compared with US is the dexterity of using two “free” hands for needle advancement, which is not possible in US where one hand is restricted to transducer support and manipulation. Interestingly, Battaglia and colleagues considered the presence of two operators to be at the base of a successful US-guided aspiration—one maintaining needle position and aspiration while the second holding the probe for real-time evaluation [17], but we deem this to be a distinct disadvantage, especially in centers with limited resources. Overall, while US can perform well in the setting of an enlocated THA, its viability in the setting of AD-THA has not been investigated, and further studies, including a comparison to fluoroscopy guidance, are necessary.

In comparison to fluoroscopy, computed tomography (CT) exhibits a superior depiction of cross-sectional anatomy which may theoretically make it an effective modality for aspiration of AD-THA. Aside from providing a sample for microbiologic studies, CT shows periprosthetic collections and enlarged regional lymph nodes which are significant predictors of PJI [21], while also depicting critical hardware and osseous integrity or disruption. There is no previous data available on cost, length of procedure time, and radiation dose specific to THA aspirations performed under CT compared with conventional fluoroscopy, although deducing from studies performed on other non-aspiration procedures [14, 22–25], these parameters may be higher compared with conventional fluoroscopy. CT may also be limited by beam hardening artifacts resulting from the metallic hardware, despite metal artifact reduction protocols [26]. There is currently no study comparing fluoroscopy and CT imaging modalities to one another whether aspirating an enlocated or dislocated THA.

This study is limited by small sample size, although we believe it provides important initial information given that there is no previous study on image-guided aspiration of AD-THA. There may also be variability in the technique used given the different experience and expertise levels of each operator. This variation in training could represent differences in each operator’s ability to obtain spontaneous aspirations and fluoroscopic time. The aspirations were all performed on adults, and therefore, the findings of our study may not be applicable to the pediatric population. Lastly, body mass index (BMI) was not available in all patients. Increased BMI has been shown to be an independent risk factor in reducing the likelihood of a successful aspiration [27] and increases fluoroscopy times during fluoroscopic-guided injections [28].

## Conclusion

Fluoroscopic-guided aspiration of AD-THA is a feasible, high-yield, and safe procedure for radiologists to perform for evaluation of PJI. We recommend targeting the acetabular cup as it results in higher rate of spontaneous aspiration, larger aspiration volumes, and lower fluoroscopy time compared to the femoral prosthesis.

## Data Availability

The data can be obtained from the first author or corresponding author.

## Abbreviations

AD-THA: Acutely dislocated total hip arthroplasty
CT: Computed tomography
PJI: Prosthetic joint infection
SD: Standard deviation
THA: Total hip arthroplasty

## Materials availability

The material can be obtained from the first author or corresponding author.
