# Correction: Fluoroscopy-guided aspiration of the acutely dislocated total hip arthroplasty: a feasible, high-yield, and safe procedure

**DOI:** 10.1186/s13244-025-01963-1

**Published:** 2025-04-14

**Authors:** Dyan V. Flores, Abdullah Felemban, Taryn Hodgdon, Paul Beaulé, George Grammatopoulos, Kawan S. Rakhra

**Affiliations:** 1 Department of Radiology, Radiation Oncology and Medical Physics, Faculty of Medicine, University of Ottawa, Ottawa, ON Canada; 2 Department of Medical Imaging, The Ottawa Hospital, Ottawa, ON Canada; 3 Ottawa Hospital Research Institute, Ottawa, ON Canada; 4 Department of Radiology, King Faisal Specialist Hospital and Research Center, Riyadh, Saudi Arabia; 5 Division of Orthopaedic Surgery, The Ottawa Hospital, Ottawa, ON Canada

**Correction to**: **Insights into Imaging**

10.1186/s13244-024-01880-9, published online 10 January 2025

In this article, the last name of author George Grammatopoulos was incorrectly written as Grammatopolous.

The original article has been corrected.

